# Immunotherapeutic Targeting of NG2/CSPG4 in Solid Organ Cancers

**DOI:** 10.3390/vaccines10071023

**Published:** 2022-06-26

**Authors:** Hongyu Zhang, Zhenyu Wu, Deyu Hu, Min Yan, Jing Sun, Jiejuan Lai, Lianhua Bai

**Affiliations:** 1 Hepatobiliary Institute, Southwest Hospital, Army Medical University, Chongqing 400038, China; hyz125@outlook.com (H.Z.); wzy6666662019@outlook.com (Z.W.); hudeyu233@163.com (D.H.); 13467112918@163.com (M.Y.); xnyysunjing@163.com (J.S.); laijiejuan@163.com (J.L.); 2 Bioengineering College, Chongqing University, Chongqing 400044, China; 3 Department of Nuclear Medicine, The First Affiliated Hospital, Shanxi Medical University, Taiyuan 030000, China

**Keywords:** antibody, chondroitin sulfate chain, immunotherapy, liver cancer, NG2/CSPG4, pancreatic cancer, stem/progenitor cells, tumor vaccine

## Abstract

Neuro-glia antigen 2/chondroitin sulfate proteoglycan 4 (NG2/CSPG4, also called MCSP, HMW-MAA, MSK16, MCSPG, MEL-CSPG, or gp240) is a large cell-surface antigen and an unusual cell membrane integral glycoprotein frequently expressed on undifferentiated precursor cells in multiple solid organ cancers, including cancers of the liver, pancreas, lungs, and kidneys. It is a valuable molecule involved in cancer cell adhesion, invasion, spreading, angiogenesis, complement inhibition, and signaling. Although the biological significance underlying NG2/CSPG4 proteoglycan involvement in cancer progression needs to be better defined, based on the current evidence, NG2/CSPG4^+^ cells, such as pericytes (PCs, NG2^+^/CD146^+^/PDGFR-β^+^) and cancer stem cells (CSCs), are closely associated with the liver malignancy, hepatocellular carcinoma (HCC), pancreatic malignancy, and pancreatic ductal adenocarcinoma (PDAC) as well as poor prognoses. Importantly, with a unique method, we successfully purified NG2/CSPG4-expressing cells from human HCC and PDAC vasculature tissue blocks (by core needle biopsy). The cells appeared to be spheres that stably expanded in cultures. As such, these cells have the potential to be used as sources of target antigens. Herein, we provide new information on the possibilities of frequently selecting NG2/CSPG4 as a solid organ cancer biomarker or exploiting expressing cells such as CSCs, or the PG/chondroitin sulfate chain of NG2/CSPG4 on the cell membrane as specific antigens for the development of antibody- and vaccine-based immunotherapeutic approaches to treat these cancers.

## 1. Brief Introduction to NG2/CSPG4 in Solid Organ Cancers

**Figure 1:**
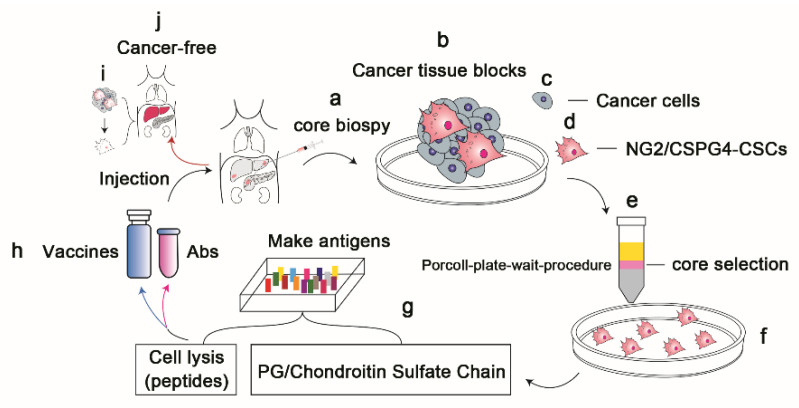
A novel strategy for developing vaccine- or antibody-based therapeutic approaches to target NG2/CSPG4-expressing membrane or cancer stem cells (CSCs) in solid organ cancers. (**a**,**b**) Core needle biopsies (**a**) and obtained tissue blocks (**b**) from solid organ (liver, pancreas, lungs, or kidneys) cancers. (**c**,**d**) The obtained cancer tissue blocks contained both cancer cells (**c**) and NG2/CSPG4-expressing cells (**d**), defined as CSCs. (**e**,**f**) Use of a unique method, the “Percoll−Plate−Wait procedure”, as the core selection method to purify NG2/CSPG4-expressing cells from the blocks and expand them in cultures (**f**–**j**) Use of the purified NG2/CSPG4-expressing cells to produce different antigen types, either cell lysates (peptides) or PG/chondroitin sulfate chain membranes (**g**), as vaccine or antibody targets (**h**) to eliminate NG2/CSPG4-expressing cells (such as CSCs). The cancer mass ultimately regressed (**i**), creating a population of cancer-free patients that received (injection) vaccines or antibodies (**j**).

Forty years ago, NG2/CSPG4 proteoglycan (PG) was discovered as a marker of oligodendrocyte precursor cells (OPCs), together with platelet-derived growth factor receptor á (PDGFR-á) in the developing central nervous system (CNS) [1] and CNS cancers such as gliomas [2]. Since then, many studies have demonstrated that NG2/CSPG4 is also a marker of immature cell types outside the CNS [3,4], which correlates with solid organ malignancies, such as cancers of the liver, pancreas, lungs, and kidneys [5,6,7]. In recent years, progress has led to the NG2/CSPG4 molecule being defined as a novel prognostic indicator in human solid organ cancers, such as hepatocellular carcinoma (HCC) [5]. It is expected that there will be an increase in the pace of our acquisition of knowledge on the role played by NG2/CSPG4 in solid organ malignancies in the future. Herein, we attempt to dissect a new perspective on the expression of NG2/CSPG4 in solid organ cancers, such as HCC, pancreatic ductal adenocarcinoma (PDAC), and cancers of the lungs or kidneys, and its clinical significance as a novel immunotherapeutic target (Figure 1).

## 2. Initial Discovery of NG2/CSPG4 as a Precursor Marker in the CNS and Its Association with CNS Cancers

Concerning the identification of the NG2/CSPG4 molecule as a serological epitope, this molecule was first reported to be present on a subpopulation of neural cell lines and was initially characterized with a sequentially absorbed rabbit antiserum; then, the epitope was prepared for monoclonal antibody (mAb) recognition [8]. Two years later, NG2/CSPG4 was identified as a large molecule derived from the >300 kDa chondroitin sulfate PG [9]. This polypeptide contains numerous glycosylation sites and three putative glycosaminoglycan (GAG)-attachment sites. Owing to its extended extracellular domain, NG2/CSPG4 possesses the potential to engage in a multitude of molecular interactions, spanning from the sequestration of growth factors, such as basic fibroblast growth factor (bFGF) and PDGF-AA [10], signaling molecules [11], metalloproteinases (MMPs) [12], and collagens (such as IV) of the extracellular matrix (ECM) [13,14], to the binding of these ligands to cell surface receptors and the ECM. Based on the fact that the PG of NG2/CSPG4 can be extracted from cell lines, the PG was concluded to be an integral membrane component. In addition, independent studies have since identified the serological epitope on human melanoma cells [15], which is known as the high molecular weight melanoma-associated antigen (HMW-MAA), and this PG has been also designated as AN2 because of the mouse homolog of the rat PG of NG2 or transcripts for excitatory amino acid carrier 1, a neuronal glutamate transporter [16]. Throughout this review, we will refer to these molecules collectively as NG2/CSPG4.

The expression pattern of NG2/CSPG4 has always been of interest and the ability to use NG2/CSPG4 as a marker for unique cell types is a strong motivation for us. Studies have indicated that NG2/CSPG4-expressing cells are precursors, as NG2/CSPG4 is characteristic of immature neural cell lines capable of differentiating into either glia or neurons (the name implies nerve/glial antigen 2, “NG2”), which seems plausible as most immature cell lines are derived from cancers that were induced in animal embryos [17] where many immature precursors are expected to exist. NG2/CSPG4 expression on immature cell types occurs prior to differentiation into the associated mature cell types, indicating that NG2/CSPG4 is a valuable marker for progenitor cells in the embryonic CNS [18].

It has also been reported that a large number of NG2/CSPG4-expressing cells, as progenitors present in the adult CNS [1], play a major [19,20] or supporting role [21]. However, due to technique limitations related to the isolation of NG2/CSPG4-expressing cells from adult solid organ tissues, not excluding the CNS, the question of whether NG2-expressing cells in the adult CNS operate in the same manner as those in the embryonic CNS has yet to be resolved. These questions were addressed by Bai L et al., who used a “Percoll−Plate−Wait procedure”, a unique method used to successfully isolate NG2/CSPG4-expressing cells from multiple adult solid organ tissues [22] including the CNS, and demonstrated that these NG2/CSPG4-expressing cells act as stem/progenitor cells in injured tissue repair [23,24,25,26,27]. Additionally, synaptic stimulation of cells in response to a synaptic input [21] confirmed NG2 as a marker for progenitors of unique adult cell types.

NG2/CSPG4 is a malignant marker in CNS cancers such as gliomas, the most common and malignant cancer in the CNS [28], and it is closely related to poor prognoses as it promotes cancer cell proliferation and motility via its binding to growth factors (bFGF and PDGF-AA) [24], MMPs [29], ECM-collagen IV [30], and integrins [31], which involves these binding partners in an oncogenic transformation. There is an inverse relationship between the activation of á3â1 integrin-dependent PI3K/Akt signaling promoted by NG2/CSPG4 and apoptosis, as demonstrated by the restoration of apoptosis after siRNA knockdown of NG2/CSPG4 [32]. Furthermore, NG2/CSPG4-expressing cells are the most important population of cycling cells in the adult CNS and gene mutations can accumulate, leading to glioma tumorigenesis [33,34]. As a whole, all these observations are in line with most gliomas originating from the subcortical white matter rich in immature cells expressing NG2/CSPG4 [35].

## 3. NG2/CSPG4 as a Marker of Precursor or Progenitor Cells outside the CNS and Its Association with Solid Organ Cancers

The solid organs include the liver, pancreas, heart, lungs, and kidneys. Far from being restricted to acting as a marker of progenitor cells related to CNS physiology and tumorigenesis, NG2/CSPG4 is widely expressed outside the CNS in multiple solid organ tissues [36] by a variety of mesenchymal cell types [37,38].

In the physiological developmental pattern, NG2/CSPG4 expression is maintained in only a relatively developmentally restricted population of partially differentiated progenitor cells that have made an initial commitment to a particular lineage. These restricted NG2/CSPG4-expressing progenitor cells are mitotic and likely to retain a degree of developmental plasticity. When they undergo terminal differentiation and become quiescent, NG2/CSPG4 expression is downregulated [39]. Many studies have focused on NG2/CSPG4-expressing pericytes (PCs) [40] in the developing vasculature as an angiogenic marker of an activated vasculature status [41] because most studies have convincingly demonstrated that NG2/CSPG4 is expressed on the surface of vascular mesenchymal/mural cells [42] but not on that of vascular endothelial cells (ECs) [43]. In the physiological adult pattern, NG2/CSPG4 is present in multiple adult organ tissues [22]. For example, in regard to vascular mural progenitor cells, NG2/CSPG4 is expressed on heart cardiomyocytes in large vessels, specifically smooth muscle cells (SMAs) [44], and on PCs in the microvasculature [45], making it even more meaningful for heart disease [46] than it has been in studies on the CNS. Based on the vital lessons learned from studies showing that NG2/CSPG4 is expressed in the vasculature of other adult organs, such as the liver [23,47], pancreas [48], lungs [49], kidneys [7], eyes [50], skin [51], and bone marrow [52], it is possible that the blood vessel walls harbor a reserve of NG2/CSPG4-expressing PCs as progenitor cells that may be integral to the elusive origin of mesenchymal stem cells (MSCs) [36,52,53] and other related adult solid tissue stem cells (SSCs).

**Figure 2:**
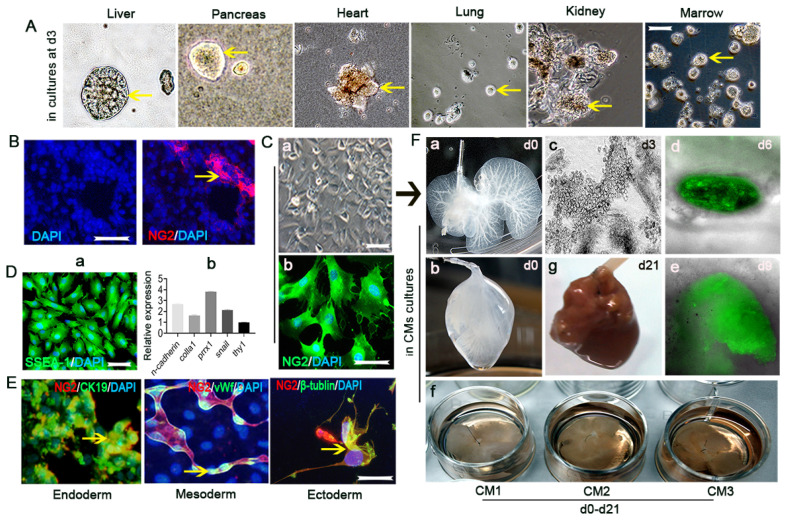
The novel method of “Percoll−Plate−Wait procedure” for NG2/CSPG4-expressing cell isolation from multiple adult mouse solid organs. (**A**) NG2/CSPG4-expressing cells were isolated from the adult liver, pancreas, heart, lungs, kidneys, or bone marrow and cultured as spheres (arrows). Scale bar = 100 µm. (**B**) Hepatic NG2/CSPG4-expressing cells (red, denoted with an arrow) are located in the portal perivascular area (denoted with a box). Scale bar = 100 µm. (**Ca**,**b**) Immunostained the cells in cultures (**a**) isolated by our supplementary Information mentioned protocol exhibit 95–98% NG2-positive (green, **b**). Scale bar = 100 µm. ((**Da**,**b**),**E**,**F**) The isolated NG2/CSPG4-expressing cells showed embryonic stem cell-like features: (**Da**) expression of SSEA-a at the protein level, identified by immunostaining (green, scale bar = 100 µm); (**Db**) expression of n-cadherin, colla-1, snail, and thy-1 at the mRNA level, identified by RT–qPCR; transgerm differentiation including CK19 (endoderm), vWf (mesoderm), and β-tublin (ectoderm) ((**E**), denoted with arrows); (**Fa**–**f**)) NG2^+^ cells as pluripotent seed cells re-cellularized into de-cellularized liver scaffold (**a**,**b**), in 37 °C cultures (**f**), gradually formed live-like tissues (**c**–**e**) and liver-like organ finally after 21 days of culture (**Fg**) in different conditioned mediums (CMs). CMs consist of DMEM/F12 and neonatal mouse liver homogenate as 4:1, including VEGF etc., (CM1 for first week), bFGF, insulin etc., (CM2 for second week), and HGF etc., (CM3 for last week).

Based on our recent studies, NG2/CSPG4-expressing cells isolated from multiple adult solid organs (the liver, pancreas, heart, lungs, and kidneys) including bone marrow by using the “Percoll−Plate−Wait” procedure method [23] exhibit characteristics of embryonic-like stem cells. For example, with the protocol [23], the multiple organ source NG2/CSPG4-expressing cells with spheres (arrows) developing in vitro (Figure 2A), the liver portal triad source NG2/CSPG4-expressing selected clone cells (red, an arrow, Figure 2B) [23] with more than 95% of NG2/CSPG4 glycoprotein (Figure 2(Cb)) [23] clone expand in cultures (Figure 2(Ca)) [23], strongly positive for stage-specific embryonic antigen (SSEA-1) (Figure 2(Da), by immunostaining, green), an embryonic marker of mRNA expression of *n-cadherin*, *colla1*, *prrx1*, *snail* and *thy1*, the totipotent genes (Figure 2(Db) identified by quantitative reverse transcription-PCR, RT–qPCR) [54]. Additionally, this embryonic-like property of the liver portal triad source NG2/CSPG4-expressing cells is also reflected in the transgerm layer differentiation potential aspect when culturing them in special conditions (Figure 2E) and the liver organ construction potential aspect in conditioned mediums (CMs), including VEGF containing CM1 when the culture was starting (d0–7), bFGF, insulin containing CM2 during the second week (d7–14) and HGF containing CM3 during the last week (d14–21) when re-cellularized into a de-cellularized bioscaffold (Figure 2(Fa–g)) [55]. Therefore, we speculated that it would be possible to infer the possible type of embryonic-like stem cell population that remains in adult organ tissue following embryonic development to participate in adult injured tissue repair and regeneration, which is currently being studied.

There is scant information regarding the glycosylation/glycanation patterns of NG2/CSPG4 molecules expressed by the solid organ cancer vasculature or how these patterns may be related to the structural−functional diversities of this PG [55,56]. It would be of considerable interest to understand the biological differences in NG2/CSPG4 among physiological, pathological, and cancerous conditions and the effects of NG2/CSPG4-expressing cells on solid organ health (homeostasis), disease development, and cancerous cell shift [5]; the unequivocal experimental proof is currently lacking. Furthermore, heterogeneity in NG2/CSPG4-expressing cells [23] may account for their different tasks in solid organ tissue homeostasis and repair or regeneration that are responsible for the unregulated growth of solid organ cancers. Furthermore, NG2/CSPG4-expressing cells as PCs, also known as mural cells [57,58], are important cellular components not only in the normal blood vasculature but also in the progression of solid organ cancers, such as pancreatic cancer [59] and lung cancer [60]. Based on the finding that in the developing CNS, ECs are guided by migrating PCs during the organization of the growing vessel wall [61] and on experimental evidence showing that NG2/CSPG4-expressing PCs are more abundant than MSCs, we concluded that NG2/CSPG4-expressing MSC-like PCs become progressively upregulated with increasing malignancy in solid organ cancers, such as lung cancer [62], so it seems likely that NG2/CSPG4-expressing PCs in solid organ cancers are relatively closely related to angiogenesis [63] and metastasis [64,65].

**Figure 3:**
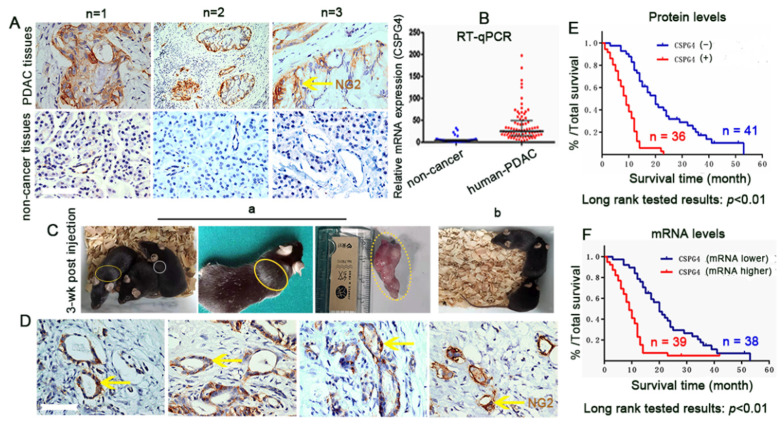
NG2/CSPG4 overexpression in pancreatic ductal adenocarcinoma (PDAC) predicts a poor prognosis. (**A**,**B**) 3,3′-Diaminobenzidine (DAB) staining (brown) and RT–qPCR for NG2/CSPG4 identification in PDAC patient tissues (top panel) and non-cancerous pancreatic tissues (low panels) at both the protein (**A**) and mRNA (**B**) levels, respectively. Scale bar = 100 µm. (**Ca**,**b**) Tumorigenesis of human hepatocellular carcinoma (HCC) cell lines (H_22_) in C56BL/6 mice injected with the NG2-isolated (NG2^+^/H_22_) cell line. In total, two (indicated by an yellow oval and a white circle) out of three mice developed tumors two weeks after cell injection (**Ca**), while control mice that received non-NG2-isolated cell lines (H_22_) (**Cb**) remained cancer-free at the same time. (**D**) The expression of NG2/CSPG4 was closely related to the intra-vessel space. Scale bar = 100 µm. (**E**,**F**) Overexpression of NG2/CSPG4 in PDAC at both the protein (**E**) and mRNA (**F**) levels predicted poor survival.

To understand the relationships between NG2/CSPG4 expression and angiogenesis or metastasis in solid organ cancers, represented by liver and pancreatic cancers, we collected both HCC and PDAC specimens from patients, respectively. A total of 132 human HCC specimens were analyzed [5]. The protein and mRNA levels of NG2/CSPG4 in HCC samples were higher than those from adjacent non-cancerous tissue [5]. Similarly, in 86 human PDAC specimens, 3,3′-Diaminobenzidine (DAB) staining (brown) identified dramatic expression of NG2/CSPG4 in PDAC tissues (Figure 3A, upper panels, arrow) compared to non-cancerous control tissues (Figure 3A, lower panels), and the mRNA expression pattern showed a similar trend (Figure 3B). Interestingly, in an experiment where inbred C57BL/6 mice were injected with HCC cell lines (H_22_), we observed more carcinogenesis (2 out of 3, denoted as a yellow oval and a white circle) in the C57BL/6 mice that received the higher NG2/CSPG4-expressing H_22_ two weeks post-cell injection (Figure 3(Ca)) than those that received the parental control cell line at the same time (Figure 3(Cb), cancer-free, Supplementary information). Focusing on the blood vessels in these two types of samples, we noted that consistently with the HCC samples [5], higher expression of NG2/CSPG4 was also more closely associated with the blood vasculature in PDAC (Figure 3D, brown, arrows) and correlated with a poor prognosis. Moreover, investigations for mortality analysis revealed higher mortality related to both the protein (Figure 3E) and the mRNA levels (Figure 3F), similar to the HCC pattern [5]. This remarkable upregulation of NG2/CSPG4 in human HCC and PDAC remains puzzling and a clear explanation has not been found; however, this may suggest that NG2/CSPG4 is able to drive metastasis. Due to technical limitations in the field, researchers have been unable to determine whether NG2/CSPG4-overexpressing cells in vasculature lesions or perivascular niches are actually correlated with the cells fated to give rise to metastases. For example, it is unclear whether malignant cells forming metastases simply upregulate NG2/CSPG4 in a site-specific manner; it is also unknown whether the upregulation of NG2/CSPG4 is a tertiary tumorigenic event and whether that significance needs to be investigated. On the other hand, our clinical findings in HCC and PDAC at least suggest that the upregulation of NG2/CSPG4 is associated with poor prognoses in these solid organ cancers and could be useful as an additional prognostic marker to increase the resolution of traditional approaches.

**Figure 4:**
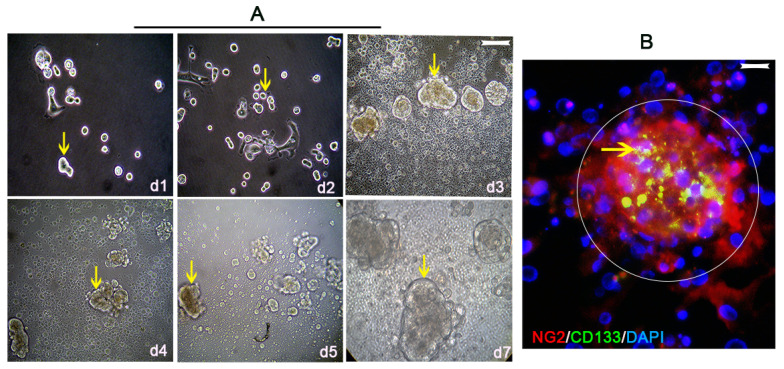
NG2/CSPG4-expressing cells purified from human PDAC tissues by the “Percoll−Plate−Wait procedure” method exhibit stem cell characteristics. (**A**) The “Percoll−Plate−Wait procedure”obtained NG2/CSPG4-expressing cells from PDAC by core biopsies that appeared as spheres (yellow arrows) and aggressively grew in cultures during the time from day 1 to day 7. Scale bar = 100 µm. Double staining for NG2 (red a sphere in a circle) and CD133 (green), a stem cell marker, revealed co-staining ((**B**), merged, denoted with an arrow, scale bar = 200 µm).

**Figure 5:**
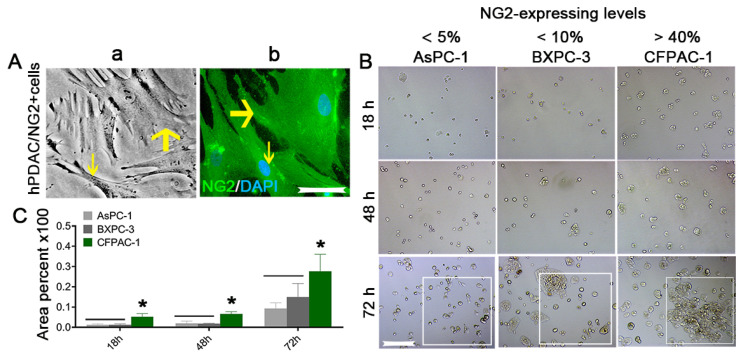
Heterogeneity and proliferative potential of isolated NG2/CSPG4^+^-expressing cells (Figure 1, NG2 expression > 40%). (**Aa**,**b**) Exhibited heterogeneity (denoted as thin and bold arrows) of NG2/CSPG4-expressing cells freshly isolated from human PDAC tissues in primary cultures (**Aa**) and immunofluorescence staining (**Ab**). Scale bar = 200 µm. (**B**) Three human PDAC cell lines with different NG2 expression levels showed different growth potentials in cultures, as identified by a CCK8 assay. Scale bar = 100 µm. (**C**) Quantification of the proliferation of the three cell lines. At least three independent experiments were performed, and all data are presented as the mean ± SD. * p < 0.05 vs. the BXPC-3 and AsPC-1 cell lines.

CSCs are known to generate vascular PCs and these cells may actively remodel perivascular niches [66,67]. We speculated that if the PCs in perivascular niches can be isolated and expanded in vitro, they will be a valuable marker for specific antibody and vaccine targets to disrupt the neovasculature created by PCs for cancer elimination. To this end, we used fresh human PDAC tissues obtained by core needle biopsy with the protocol described above [23] and successfully expanded NG2/CSPG4-expressing cells as aggressively sphere grown during the time (Figure 4A). Double immunostaining further identified that the NG2/CSPG4-expressing spheres (red) positively co-stained for CD133 (green) (Figure 4B, merged, denoted as an arrow in a circle), strongly favoring the conclusion that solid organ cancer (PDAC)-generated PCs are CSCs. Furthermore, as serum-containing cultures representing intra-blood vasculature lesions of human PDAC-isolated NG2/CSPG4-expressing cells both in culture (Figure 5(Aa)) and in immunostaining procedures (Figure 5(Ab), green) revealed obvious heterogeneity (denoted as thin and bold arrows), we speculated that the NG2/CSPG4-expressing cells pertained to aggressive cancer cell subsets; however, to date, no correlation studies have been published to clarify whether the heterogeneity of NG2/CSPG4-expressing cells within a primary or secondary cancer lesion discriminates patient subgroups with diverse clinical courses. We performed an experiment that at least suggests that the heterogeneity of NG2/CSPG4-expressing cells correlated with malignant growth. In this experiment, we selected three human PDAC cell lines (BXPC-3, AsPC-1, and CFPAC-1) with different expression levels of NG2 detected by immunostaining (BXPC-3, <5%; AsPC-1, <10%; and CFPAC-1, >40%). The cell growth of the CFPAC-1 cell line was better than that of the other two cell lines (Figure 5B). The results of a CCK8 assay (Supplementary information) showed a similar trend: the higher NG2-expressing cell line (CFPAC-1) correlated with more aggressive proliferation than the other two cell lines (BXPC-3 and ASPC-1) at all time points (Figure 5C). Interestingly, recently, when we purified NG2-expressing cells from the CFPAC-1 cell line (NG2^+^/CFPAC-1) and subcutaneously injected them into NOD/SCID mice, we observed aggressive and rapid tumorigenesis compared to the injection of parental non-purified control cells (NG2^−^/CFPAC-1, unpublished work); this may highlight the potential of selectively targeting NG2/CSPG4-expressing cells, whether as PCs within the neovasculature or as CSCs for novel isoform-specific antibody- or vaccine-based therapeutic strategies, against solid organ cancers, such as HCC and PDAC.

## 4. Selection of NG2/CSPG4 as a Target Antigen for the Treatment of Solid Organ Cancers

Due to the theranostic value of its almost exclusive expression in solid organ cancer patients, NG2/CSPG4 is an attractive candidate target for anti-NG2/CSPG4 mAb- and vaccine-based therapies.

Anti-NG2/CSPG4 mAbs have been shown to inhibit solid organ cancer progression by blocking ligand access to the extracellular binding sites of NG2/CSPG4. Therefore, NG2/CSPG4-directed antibody conjugates are selectively internalized by NG2/CSPG4-expressing cancer cells through endocytosis [68]. Additionally, based on the selective NG2/CSPG4 upregulation observed in solid organ cancer-associated PCs in intra-blood vasculature lesions, this approach may also contribute to solid organ cancer regression via the inhibition of neoangiogenesis [69]. Furthermore, evaluation of a large panel of mAbs against human NG2/CSPG4 for the generation of single-chain NG2-CSPG4/CD3-bispecific antibodies, such as bispecific T-cell engagers (BiTEs) [70], showed that binding to the membrane proximal domain D3 of NG2-CSPG4 was more potent than that to distal domains; these observations were made on the CHO cell line that expresses small surface target antigens and is generally better lysed than cells expressing larger target antigens [71], suggesting that antigen size plays a role in determining target potency.

Moreover, the NG2/CSPG4 antigen may provide the basis for the selection of maximally potent, BiTE-redirected immune cells that may be candidates for the development of a novel immunotherapeutic strategy for solid organ cancers; for example, this antigen may be useful for chimeric antigen receptor (CAR) T-cell selection [72,73,74]. Genetic engineering technologies can redirect T lymphocytes, such as CD8^+^ cytotoxic T lymphocytes (CTLs), to recognize or target a wide variety of cancer antigens via the expression of a CAR [75]. As NG2/CSPG4 has not been reported to be a target antigen in liver, pancreas, lung, or kidney cancers, direct immunotargeting of the NG2/CSPG4 antigen may latently arrest cancer growth and dissemination. Choosing the PG-chondroitin sulfate chain as the putative target of CAR-T cells to allow the targeting of NG2/CSPG4-expressing cell membrane microdomains by targeted therapies may be a selective and powerful approach to eliminate cancers. Therefore, the chondroitin sulfate chain [76,77] as a surface antigen derived from NG2/CSPG4 on the cell membrane should be a focal point for further developing immunotherapeutic approaches exploiting this PG subchain (PG-subChain) in solid organ cancers, such as HCC and PDAC. Except for those target antibodies or vaccines, using microarray analyses to evaluate NG2/CSPG4 expression in solid organ cancers is also compelling (Table 1). According to our new method, NG2/CSPG4-expressing cells can be isolated not only from normal adult solid organ tissues [22] but cancerous ones as well, and these expanded cultures could better support studies on novel immunotherapies targeting membrane NG2/CSPG4 either with antibodies or vaccines. Additionally, the success of antibodies targeting programmed cell death protein 1 (PD-1) and its ligand L1 (PD-L1) in solid organ cancer treatment and the need for improving response frequency could lead to an increased demand for the development of combination therapies with anti-PD-1/PD-L1 blockers as a backbone [78,79,80,81].

## 5. Conclusions and Perspective

NG2/CSPG4 was originally demonstrated to be a key player in CNS development and CNS cancers. It participates in angiogenesis and tumorigenesis, and its role as a therapeutic target has been emphasized. Expansion of the study of NG2/CSPG4 outside of CNS cancers in recent decades has produced important experimental results. However, there are too few studies on the correlations between NG2/CSPG4 biological characteristics and human solid organ homeostasis injured tissue repair and cancer formation, such as HCC and PDAC, with clinical significance. It is now expected that a novel immunotherapeutic strategy will be implemented in these human cancers. Despite the lack of broader mechanistic insights, the success of passive (antibodies) and active (CD^8+^ CTLs, the main cells exerting cytotoxic activity against cancers) anti-NG2/CSPG4 immunotargeting (vaccine) approaches are believed to provide a preclinical rationale for attempting to treat different solid organ cancers, such as liver, pancreatic, lung, and kidney cancers, through specific targeting of the overexpression of NG2/CSPG4 (PG, PG-subChain). Utilizing the autologous chondroitin sulfate chain from NG2/CSPG4-expressing cells isolated from patient solid organ cancer tissues as an antigen may highlight the greater potential of using anti-NG2/CSPG4 vaccines with finer antigen specificity than those previously evaluated.

We hope that in the near future, NG2/CSPG4 will not only remain widely utilized as a biomarker for routine clinical monitoring of patients with solid organ cancers but also be exploited as a target antigen for specific antibodies or vaccines to eliminate cancer cells.

## Figures and Tables

**Table 1:** Evaluation NG2/CSPG4 expression in solid organ cancer patients by using microcarry analysis.

Cancer Types	Patients	NG2/CSPG4		High NG2/CSPG4	References
	(Case)	mRNA	Protein	Outcomes	
Pancreas	256	qPCR	IHC *	poor	[82]
Breast	240	qPCR	IHC	poor	[83,84,85]
Breast Phyllodes	194		IHC	poor	[86]
Nead neck squcemeus carcema	unknow	qPCR	IHC	poor	[83]
Chordoma	86		IHC	poor	[87]
Glioblastema (GBM)	unknow	qPCR	IHC	poor	[88,89,90]

* Immunohistochemistry.

## Data Availability

The data presented in this study are available within the article.

## Supplementary Materials

The following are available online at https://www.mdpi.com/article/10.3390/vaccines10071023/s1, File S1: The review involved main fresh methods.

## Abbreviations

bFGF	basic fibroblast growth factor
CNS	the central nervous system
CSCs	cancer stem cells
ECs	endothelial cells
ECM	extracellular matrix
HCC	hepatocellular carcinoma
HMW-MAA	human molecular weight melanoma-associated antigen
MCSP	melanoma chondroitin sulfate proteoglycan
MMPs	metalloproteinases
MSCs	mesenchymal stem cells
NG2/CSPG4	neuro-glia antigen 2/chondroitin sulfate proteoglycan 4
OPCs	oligodendrocyte precursor cells
PCs	pericytes
PDAC	pancreatic ductal adenocarcinoma
PDGFR-á	platelet-derived growth factor receptor á
PG	proteoglycan
SMAs	smooth muscle cells
SSCs	solid stem cells
SSEA-1	stage-specific embryonic antigen
