# Supplementary material for: Immunotherapeutic Targeting of NG2/CSPG4 in Solid Organ Cancers
Source: Vaccines (Basel). 2022 Jun 26;10(7):1023. doi: 10.3390/vaccines10071023 (PMC9321363; doi:10.3390/vaccines10071023)
Supplement: Supplementary file 1 [file vaccines-10-01023-s001.zip › vaccines-1743463-supplementary.pdf]

vaccines
MDPI

Supplementary File: The review involved main fresh methods

Generation of NG2-expressing spheres

Primary cells isolated from healthy multiple adult organs and pancreatic cancers of patients with a novel percoll-fraction protocol, mostly based on our published work [23]. Briefly, for each isolation, organ tissues from two adult (>8 weeks) C57BL/6 inbred mice or human pancreatic cancer tissues (approximately 1 mg) were dissected in Modified Eagle's Medium (MEM) and chilled. Surface blood vessels were carefully removed and tissue was dissociated for 30 min. Then, the cells obtained from digested tissue were spun at 800 x *g* for 5 min for three times before layering on a Percoll gradient (30/70%, GE Healthcare,17-0891-02). Following centrifugation at 2000 rpm for 30 min, the cellular fractions were collected, washed, and cultured in sphere growth medium: Neurobasal-A medium (Gibco 10888) supplemented with 20 ng/mL EGF (Sigma E4127), B27 supplement serum-free supplement (Gibco 17504-044) - working concentration 2% (1:50 dilution) and GlutaMAX (Gibco 35050-061) - use 1 mL per 100 mL of media (1:100 dilution, 2 mM) at a density of $1x10^6$ cells/ml in a 75 cm^2 flask and grown at 37°C in 5% CO2 with a 1mL medium addition every 3d, >95% spheres express NG2.

Highly tumor formation potential of NG2-purified hepatocellular carcinoma cell line (H_{22}) by the specific percoll-fraction procedure

The NG2-expressing cells were selected from the hepatocellular carcinoma cell line (H_{22}, Porcell, cl-0341, Wuhan, China) with the same protocol (fraction-percoll procedure) and cultured in RPMI-1640 supplyment with 10% FBS and 1%P/S. Then the C57BL/6 mice were inoculated the NG2-isolated H_{22} at the back of the neck site at density of $1x10^7$/200 μL/PBS. The size of tumor formation were assessed at 10 days compared to the mice that received non-NG2-purified H_{22}.

Cell Counting Kit-8 (CCK8) assay for comparision of pancretic cancer cell proliferation potential

CCK8 assay was used to check cell viability. The review involved a comparison of proliferation of three pancreatic cancer cell lines with different NG2-expressing levels (AsPC-1<5%; BXPC-3<10%; CFPAC-1>40%) among the three groups . These cell proliferation/growth was detected by CCK-8 assay. The growth rate of CFPAC cells increased significantly compared to other two groups (shown in Figure. 6B,C).
